# The therapeutic potential of purified cannabidiol

**DOI:** 10.1186/s42238-023-00186-9

**Published:** 2023-06-13

**Authors:** Saoirse Elizabeth O’Sullivan, Sanne Skov Jensen, Gitte Nykjaer Nikolajsen, Heidi Ziegler Bruun, Rhenu Bhuller, Julia Hoeng

**Affiliations:** 1 CanPharmaConsulting, Nottingham, NG9 3BB UK; 2 Fertin Pharma, Dandyvej 19, Vejle, 7100 Denmark; 3 Vectura Fertin Pharma, Basel, Switzerland

**Keywords:** Cannabidiol, CBD, Cannabinoid, Clinical trial, Evidence, Neurology

## Abstract

The use of cannabidiol (CBD) for therapeutic purposes is receiving considerable attention, with speculation that CBD can be useful in a wide range of conditions. Only one product, a purified form of plant-derived CBD in solution (Epidiolex), is approved for the treatment of seizures in patients with Lennox-Gastaut syndrome, Dravet syndrome, or tuberous sclerosis complex. Appraisal of the therapeutic evidence base for CBD is complicated by the fact that CBD products sometimes have additional phytochemicals (like tetrahydrocannabinol (THC)) present, which can make the identification of the active pharmaceutical ingredient (API) in positive studies difficult. The aim of the present review is to critically review clinical studies using purified CBD products only, in order to establish the upcoming indications for which purified CBD might be beneficial. The areas in which there is the most clinical evidence to support the use of CBD are in the treatment of anxiety (positive data in 7 uncontrolled studies and 17 randomised controlled trials (RCTs)), psychosis and schizophrenia (positive data in 1 uncontrolled study and 8 RCTs), PTSD (positive data in 2 uncontrolled studies and 4 RCTs) and substance abuse (positive data in 2 uncontrolled studies and 3 RCTs). Seven uncontrolled studies support the use of CBD to improve sleep quality, but this has only been verified in one small RCT. Limited evidence supports the use of CBD for the treatment of Parkinson’s (3 positive uncontrolled studies and 2 positive RCTs), autism (3 positive RCTs), smoking cessation (2 positive RCTs), graft-versus-host disease and intestinal permeability (1 positive RCT each). Current RCT evidence does not support the use of purified oral CBD in pain (at least as an acute analgesic) or for the treatment of COVID symptoms, cancer, Huntington’s or type 2 diabetes. In conclusion, published clinical evidence does support the use of purified CBD in multiple indications beyond epilepsy. However, the evidence base is limited by the number of trials only investigating the acute effects of CBD, testing CBD in healthy volunteers, or in very small patient numbers. Large confirmatory phase 3 trials are required in all indications.

## Background

The use, acceptance and legality of cannabinoid-based medicines and the cannabinoid cannabidiol (CBD) have grown considerably in the last 10 years. CBD is in the unusual position where it is available as both a health supplement and as a licensed medicine. As a health supplement, it is estimated that ~ 10% of populations are using CBD [1]. In the UK, the recommended daily allowance of CBD is 70 mg/day in these types of products [2]. Anecdote and survey data suggest that people take CBD for anxiety, stress, depression, sleep, gastrointestinal problems and pain [3–5]. Some of the reputation of CBD’s therapeutic utility across such a broad range of conditions is based on scientific evidence, albeit still at a preclinical level.

Clinically, a purified oral solution of CBD (Epidiolex) is approved for the management of seizures in patients with Lennox-Gastaut syndrome, Dravet syndrome or tuberous sclerosis complex on the basis of multiple positive, phase 3 randomised controlled trials (RCTs) [6]. Patients typically take a dose of between 20 and 25 mg/kg/day, with an average seizure reduction of ~ 50% [7] and a mean serum concentration of ~ 125 ng/ml [8].

CBD products come in many forms; they can be purified synthetically or by plant extraction processes (usually at least ~ 98% pure CBD), broad spectrum distillates (including many of the other phytocannabinoids and phytochemicals) or full spectrum (including all the phytochemicals from the cannabis plant from which the CBD was extracted). Spectrum products are sometimes also called enriched or artisan products. CBD products can also come in defined ratios with Δ^9^tetrahydrocannabinol (THC), for example, 20:1 CBD to THC products. In any of the non-purified CBD products that have therapeutic utility, it is difficult to tell whether the benefit of the product arises from CBD as the active pharmaceutic ingredient (API), or as a consequence of the other chemicals present, or as a consequence of any interaction (pharmacodynamic or pharmacokinetic) between the compounds (referred to as the entourage hypothesis).

**Fig. 1:**
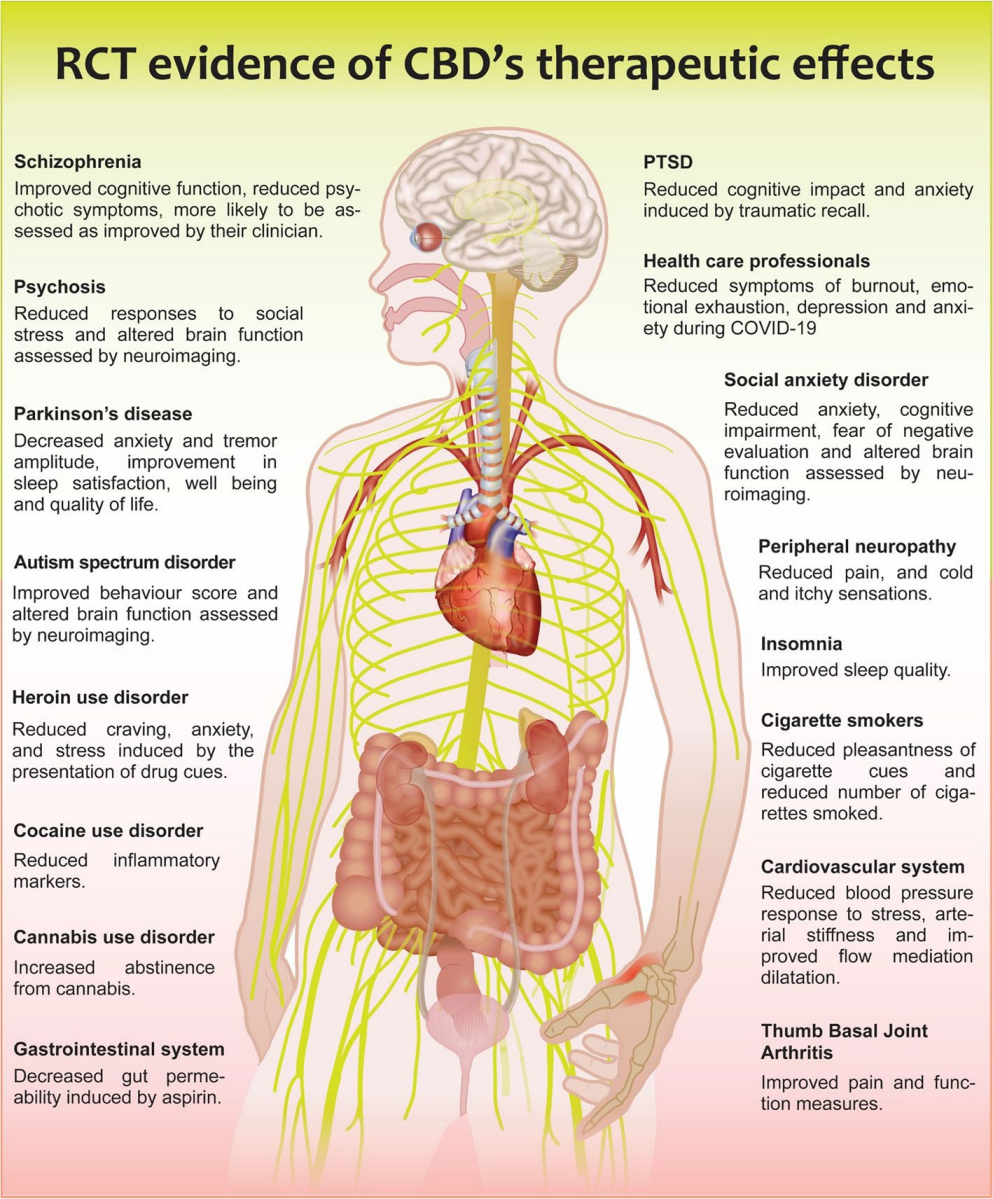
Summary of the therapeutic benefit of purified CBD across multiple conditions obtained through randomised controlled trials. PTSD, post traumatic stress disorder

The aim of this review is to establish what indications there are clinical evidence for the efficacy of purified CBD products only, without any complication of additional APIs or drug interactions. This is critical in the understanding of the CBD molecule. We have not considered epilepsy, where multiple reviews already exist, and the efficacy of purified CBD is well established [9, 10]. We addressed the aim of the study by appraising the published literature of clinical studies that have examined the therapeutic benefit of purified CBD in various conditions. A total of 99 clinical studies testing purified CBD were identified through systematic searching of engines and hand searching; 29 were uncontrolled/observational studies, and 70 were randomised controlled trials (RCTs). These studies were categorised by disease indication and are discussed below in the order of the weight of evidence/number of studies per condition. A summary of the patient cohorts in which CBD has clinical evidence of significant efficacy through controlled studies is presented in Table 1 and Fig. 1.

**Table 1:** Randomised, controlled clinical studies showing a significant effect of purified CBD in patients and in healthy volunteers. Studies are divided by the patient cohort in which CBD was tested and the treatment regime. Where measured, plasma levels of CBD are reported. Abbreviations: n/a, not available; nM, nanomolar; PTSD, post traumatic stress disorder; REM, rapid eye movement; THC-COOH, 11-Nor-9-carboxy-Δ9-tetrahydrocannabinol; Δ ^9^-THC, Δ9-tetrahydrocannabinol

Patient cohort/ indication	Treatment regime	Endpoint modified	Plasma level of CBD	Ref
Schizophrenia	800 mg/day for 6 weeks	Improvements from baseline in processing speed, visual memory, visuomotor coordination and sustained attention	n/a	[11]
	800 mg/day for 4 weeks	Improvements in Brief Psychiatric Rating Scale or Positive and Negative Syndrome Scale scores on day 14 or day 28	n/a	[12]
	1000 mg/day for 6 weeks	Lower levels of psychotic symptoms (Positive and Negative Syndrome Scale) and more likely to be assessed by their treating clinician as clinically improved and/or not severely unwell	n/a	[13]
Psychosis	600 mg, single dose	Attenuated dysfunctions in prefrontal and mediotemporal brain regions Modulation of medial temporal and striatal function during fear processing Attenuation of activation in the left insula/parietal operculum during motivational salience processing Modulation of striatum, medial temporal cortex and midbrain regions activation during a verbal learning task	~ 250 nM after 270 min	[14–17]
	600 mg for 8 days	Reduced cortisol reactivity and psychological response to social stress	n/a	[18]
PTSD	300 mg, single dose	Attenuated cognitive impact induced by the recall of traumatic events Reduced anxiety induced by the recall of traumatic events in patients with nonsexual trauma	n/a	[19, 20]
Health care professionals during COVID-19	300 mg/day for 28 days	Reduced symptoms of burnout, emotional exhaustion, depression and anxiety in health care professionals working with patients during COVID-19	~ 57 nM on day 21	[21]
Parkinson’s disease	300 mg, single dose	Decreased anxiety in response to public speaking and decreased tremor amplitude in an anxiogenic situation	n/a	[22]
	300 mg for 14 weeks	Improvement in sleep satisfaction from the 4th to 8th week	n/a	[23]
	300 mg/day for 6 weeks	Improvement in well-being and quality of life	n/a	[24]
	300 mg/day for 14 weeks	No change in REM sleep behaviour disorder but an improvement in sleep satisfaction	n/a	[23]
Social anxiety disorder	400 mg, single dose	Decreased subjective anxiety, reduced ECD uptake in the left parahippocampal gyrus, hippocampus and inferior temporal gyrus	n/a	[25]
	600 mg, single dose	Reduced anxiety, cognitive impairment, and discomfort to public speaking	n/a	[26]
	300 mg for 4 weeks	Reduction in Fear of Negative Evaluation scores and Scores of Liebowitz Social Anxiety Scale	n/a	[27]
Autism spectrum disorder	600 mg, single dose	Altered regional fractional amplitude of low-frequency fluctuations and functional connectivity in/between cerebellar vermis, the right fusiform gyrus, subcortical (striatal) and cortical targets Increased glutamate in the basal ganglia and decreased it in the dorsomedial prefrontal cortex. Decreased GABA + in prefrontal and subcortical regions	n/a	[28, 29]
	1200 mg/day for 8 weeks	Greater improvement in the Aberrant Behaviour Checklist score	n/a	[30]
Insomnia	160 mg, single dose	Improvement in subjective sleep quality	n/a	[31]
Thumb basal joint arthritis	Topical: 6.2 mg/mL CBD for 2 weeks	Improved patient-reported outcome measures (VAS pain, disabilities of the arm, shoulder, and hand; single assessment numeric evaluation)	n/a	[32]
Peripheral neuropathy	Topical: 250 mg CBD/3 fl. Oz for 4 weeks	Reduction in intense pain, sharp pain, cold and itchy sensations	n/a	[33]
Heroin use disorder	400 or 800 mg for 3 days	Reduced both craving and anxiety, cortisol and heart rate induced by the presentation of salient drug cues	n/a	[34]
Cocaine use disorder	800 mg for 92 days	Reduction in inflammatory markers after detoxification	~ 245 nM at week 12	[35]
Cannabis use disorder	400 mg or 800 mg for 4 weeks	Reduced urinary THC-COOH to creatinine concentrations and increased abstinence from cannabis	n/a	[36]
Cigarette smokers	800 mg, single dose	Reduced the salience and pleasantness of cigarette cues and reduced explicit pleasantness of cigarette images	n/a	[37]
	400 µg per inhalation for 7 days	Reduced the number of cigarettes smoked by ~ 40%	n/a	[38]
Healthy volunteers	1 mg/kg or 60 mg, single dose	Blocked the anxiety and subjective alterations induced by Δ ^9^-THC		[39, 40]
	300 mg, single dose	Reduced anxiety in response to public speaking	n/a	[41–43]
	32 mg inhaled single dose	Enhanced consolidation of explicit fear extinction	n/a	[44]
	400 mg, single dose	Decreased subjective anxiety and increased mental sedation with changes in the limbic and paralimbic brain areas	n/a	[45]
	600 mg, single dose	Disruption of prefrontal-subocritical connectivity during emotional processing Attenuated the blood oxygenation level-dependent signal in the amygdala and the anterior and posterior cingulate cortex while subjects were processing fearful faces Reduced resting blood pressure and blood pressure responses to stress Decreased gut permeability induced by aspirin	~ 47 nM at 2 h	[46–48]
	600 mg, 7 days	Reduced blood pressure response to stress, increased internal carotid artery diameter, reduced arterial stiffness and improved flow mediation dilatation	n/a	[49]

## Purified CBD as an anxiolytic

The greatest number of studies that have investigated purified CBD have been in the area of reducing anxiety. There is strong preclinical evidence to support CBDs anxiolytic effects in multiple animal models, mediated by a number of mechanisms including 5-HT1, 5-HT3, CB_1_, GPR3 and GPR6, GABA and PPARγ (for reviews, see [50, 51]).

Six uncontrolled clinical studies have reported on the efficacy of CBD in anxiety. Four case reports document that CBD was successful at reducing anxiety symptoms in teenagers/young adults with substance abuse, social anxiety and depression [52], social anxiety and psychotic symptoms [53], cannabis withdrawal syndrome [54] and Crohn’s disease with social phobia [55]. Similarly, two case series report that CBD reduced symptoms of anxiety in several hundred patients prescribed CBD via clinics [56, 57]. Most recently, Berger and colleagues report that 12 weeks treatment with purified CBD (up to 800 mg/day) led to a significant reduction in anxiety and depression in an open label study of 30 patients with anxiety disorders [58].

Since the 1970s, researchers have been investigating the ability of CBD to reduce anxiety and stress in RCTs in healthy (mostly male) volunteers. Early studies showed that acute doses of CBD (60 mg (*n* = 40) or 1 mg/kg (*n* = 8)) could reduce the anxiety produced by THC administration [39, 40]. A series of studies went on to show that a single oral administration of CBD (300 mg) is able to reduce the acute anxiety caused by public speaking in a total of 157 volunteers (some of which received placebo or other CBD doses) [41–43]. Functional brain imaging studies in healthy (mostly male) volunteers showed that CBD (600 mg) attenuated the blood oxygenation signal in the amygdala and the anterior and posterior cingulate cortex while subjects (*n* = 15) were processing intensely fearful faces [59] and reduced prefrontal-subcortical neural connectivity when shown fearful faces (*n* = 15) [46], brain region imaging findings that could be translated into anxiolytic effects. Similarly, a single dose of 400 mg reduced anxiety in healthy males (*n* = 10) associated with neuroimaging changes in the limbic and paralimbic brain areas [45]. A single inhalation of CBD (32 mg, *n* = 48) enhanced the consolidation of extinction learning (that is, helps people to forget fearful memories), which may underpin the anxiolytic effects of CBD [44]. Acute (*n* = 9) [47] and 7 days (*n* = 26) [49] oral treatment with CBD (600 mg) also reduces resting blood pressure and the blood pressure response to mental or physical stress in healthy males. However, the three most recent studies in healthy volunteers did not report anxiolytic effects of CBD. Stanley and colleagues found that a single oral dose CBD (150, 300 or 600 mg) did not affect self-reported anxiety induced by an experimentally induced examination scenario in 32 college students (although the vehicle group had higher levels of anxiety across timepoints) [60]. It is interesting that this study was mainly in female participants, and recent preclinical data suggest that the anxiolytic effects of CBD are more pronounced in males rats and vary greatly across the female rat hormonal cycle [61]. A single oral of CBD (600 mg) also did not affect emotional processing or self-reported stress (to mental arithmetic) in healthy participants (12 male and 12 female) in a neuroimaging study [62]. And finally, a single dose of oral CBD (150, 300 or 600 mg across 61 healthy adults) did not alter the self-reported fear, panic or tachycardia induced by a 10% carbon dioxide-enriched air breathing challenge [63]. It is possible that CBD modifies some stressful/anxiogenic situations and not others, and it is worth noting that neither the examination scenario or mental arithmetic induced large increases in anxiety in the most recent studies.

Using more relevant patient cohorts, nine RCTs have assessed the anxiolytic effects of CBD, of which three were negative, and 6 were positive. Looking first at the negative studies, a single dose of CBD (600 mg) did not affect persecutory ideation and anxiety, cortisol or blood pressure in patients with high paranoid traits [64]. Ten days treatment with CBD (300 mg) did not improve indicators of anxiety, depression or sleep in 31 patients with crack cocaine dependence [65]. Weekly administration of CBD (300 mg, one per week for 8 weeks) in conjunction with a therapy session, did not enhance the therapy responses in 80 patients with social anxiety disorder and panic disorder with agoraphobia [66]. However, positive effects of CBD were observed in other patient cohorts. In treatment-naïve patients with generalised social anxiety disorder (SAD, *n* = 10), a single dose of CBD (400 mg) reduced subjective anxiety associated with changes in blood flow in limbic and paralimbic areas [25]. Similarly, a single dose of CBD (600 mg) reduced anxiety and cognitive impairment in 36 treatment-naïve SAD patients induced by public speaking [26]. Another RCT showed that 4 weeks treatment with CBD (300 mg) reduced negative evaluation and anxiety scores in teenagers with SAD (*n* = 40) [27]. In patients with heroin use disorder (*n* = 42), 3 days treatment with CBD (400 or 800 mg) reduced craving and anxiety, cortisol and tachycardia induced by the presentation of drug cues, which persisted 7 days after the final CBD exposure [34]. Oral CBD (600 mg for 1 week, *n* = 58) also reduced the anxiety induced by public speaking in patients at high risk for psychosis [18] and in 24 patients with Parkinson’s disease (one 300 mg dose [22]).

In conclusion, in healthy volunteers, there is conclusive evidence that a single dose of CBD (300–600 mg) can reduce blood pressure and anxiety associated with changes in brain activity and connectivity shown by neuroimaging. Several studies suggest CBD is effective in SAD, with mixed results on anxiety induced by substance abuse, and in anxiety comorbid to other conditions. There are a number of ongoing trials investigating CBD in SAD (NCT05571592, NCT03549819) and in anxiety associated with paediatric epilepsy (NCT05324449), advanced breast cancer (NCT04482244), anorexia nervosa (NCT04878627), bipolar disorder (NCT05457465) and Alzheimer’s disease (NCT04075435), which will add to this growing evidence base.

## Purified CBD in psychiatric conditions (psychosis, schizophrenia and bipolar disorder)

Many preclinical studies have demonstrated the role of pure CBD in ameliorating the symptoms of psychosis, and in models of schizophrenia (such as reducing stereotypy, hyperlocomotion, normalisation of social withdrawal and cognitive improvements) involving CB_1_ and CB_2_, 5-HT1A receptor and neurogenesis factors [67–69].

Zuardi and colleagues first reported in 1995 in a patient that oral CBD (up to 1500 mg/day) led to improvements in Brief Psychiatric Rating Scale (BPRS) symptoms and a diazepam dose reduction in a 19-year-old female, which worsened with discontinuation of CBD [70]. In another case series, a pattern of symptom improvement was observed with oral CBD in one patient (from 40 mg/day to a maximum dose of 1280 mg/day), with symptoms worsening after discontinuation [71]. However, two patients in the same series did not demonstrate any change in symptoms of psychosis with the same dosing regimen [71]. Two case reports assessing CBD (oral dosing from 600 mg/day to 1200 mg/day for 24 days) in patients with mania associated with bipolar disorder did not report any symptom improvement during monotherapy phases (although one patient demonstrated some improvement while on concurrent olanzapine and CBD) [72].

Several RCTs have looked at repeated CBD treatment in patients with schizophrenia. When administered as an adjunctive therapy, 1000 mg oral CBD per day reduced psychotic symptoms over a period of 6 weeks (*n* = 88), as assessed by the Positive and Negative Syndrome Scale (PANSS) and by clinical assessment [13]. Oral CBD (200 mg/day, increased to a maximum of 800 mg/day for 4 weeks) alleviated psychotic symptoms (assessed by PANSS and the Brief Psychiatric Rating Scale) after 14 days and increased serum levels of the endocannabinoid, anandamide (*n* = 42 [12]).

Evidence of CBD improving central nervous system (CNS) function or reducing CNS dysfunction in patients with schizophrenia or psychosis, or in patients at high risk of psychosis, has also been obtained through RCTs. Specific benefits include improvements from baseline with CBD in visual memory, visuomotor coordination and processing speed (200 mg/day, increased to 800 mg/day, over 6 weeks, *n* = 42 [11]) and reduced cortisol and psychological responses to social stress (600 mg/day, oral capsules for 8 days, *n* = 32 [18]). However, a single dose oral CBD capsule (300 mg or 600 mg) did not affect performance on the Stroop Color Word Test in patients with schizophrenia (*n* = 28) [73].

A series of functional neuroimaging studies have investigated the effects of a single 600 mg dose of oral CBD. CBD attenuates dysfunctions in mediotemporal and prefrontal brain regions and hippocampal-striatal functional connectivity in patients with psychosis (*n* = 34 [74]) and improves the attenuation of brain activation associated with reward processing in patients at risk for psychosis (*n* = 52 [17]). CBD also results in changes in parahippocampal gyrus and striatum activation (*n* = 52) [16]) and striatum, medial temporal cortex and midbrain regions activation (*n* = 52) [15] in untreated psychosis at-risk subjects.

In conclusion, although the data were often drawn from smaller numbers of patients, some clinical evidence exists to support the use of CBD as an adjunctive treatment in patients with schizophrenia or at high risk for developing psychosis. This is an active area of research with many registered trials ongoing investigating CBD for treatment of early psychosis (NCT04411225), psychosis comorbid to cannabis use (NCT04105231) and as a novel treatment in schizophrenia (NCT04700930, NCT02088060, NCT02926859, NCT00916201) that will continue to add to the evidence base.

## Purified CBD as a sleep aid

Anecdotal and survey data suggest that people take CBD to help improve sleep quality [3–5], and drowsiness and sleepiness are often reported as side effects of CBD medication [75]. There is a limited amount of preclinical research in this area, but a small number of studies indicate that cannabinoids modulate sleep-wake cycles [76, 77].

A number of published case reports or uncontrolled clinical trials cite improvements in sleep quality with CBD administration in patients, although only one of these was specifically in patients with sleep disorders. In an open-label study in 23 patients with epilepsy, Epidiolex (up to 50 mg/kg/day) lead to an improvement in sleep architecture recorded by electroencephalogram in 85% of e cases [78]. Another open-label study in 28 epilepsy patients found a 33% improvement in sleep duration recorded by the children’s sleep habit questionnaire after 12 months treatment with CBD [79]. Increased sleep quality has also been noted in a case report of a child with PTSD (25 mg at bedtime for 5 months [80]), in an adult with cannabis addiction (24 then 18 mg for 5 month [81]) and in 4 patients with Parkinson’s disease (75 or 300 mg/day for 6 weeks [82]). An audit [56] and a case series [57] similarly found 12% and 67% of CBD prescribed patients reported an improvement in sleep.

Three RCTs have investigated the effect of CBD with measures of sleep as primary endpoints. A study in healthy volunteers (*n* = 26) found no effect of a single dose of CBD (300 mg) in polysomnography results [83]. In a follow on to the positive effects of CBD reported in a case series in Parkinson’s, de Almeida and colleagues investigated 14 weeks treatment with CBD (75 to 300 mg/day) in 33 Parkinson’s patients with REM sleep behaviour disorder. There was no significant difference in the primary outcomes (mean nights of REM disorder/week and clinical global impression scale), but CBD was associated with an improvement in sleep satisfaction in the CBD group [23]. The only positive RCT is an early study showing that participants (*n* = 15) with sleep issues reported having slept better after receiving a single dose of 160 mg CBD compared to placebo [31].

In conclusion, although anecdote and observational studies suggest CBD improves sleep (usually comorbid to another condition), there is little RCT evidence to support this. It is not clear whether improvements in sleep are secondary to improvements in other symptoms (for example pain or anxiety), or whether there is a direct effect of CBD on sleep. Currently registered trials in this space are assessing changes in sleep disturbances comorbid to multiple sclerosis (NCT05269628) and HIV (NCT05097651).

## Purified CBD as an analgesic

There are dozens of papers investigating the analgesic effects of CBD in numerous rodent models of neuropathic and inflammatory pain, and this topic is well reviewed [84]. Preclinical data also shows that CBD can enhance the analgesic effects of opioids and attenuate opioid tolerance [85, 86]. Anecdotally, one of the primary reasons that people use CBD as a health supplement is for the relief of pain and joint pain [3–5].

In a prospective, non-randomised, open-label trial in chemotherapy-induced neuropathy (*n* = 54 patients), 8 days treatment (over the chemotherapy cycle) with 300 mg/day purified CBD led to a reduction in nerve damage (measured by vibrometry) and patient-reported cold sensitivity and swallowing/throat discomfort compared to a historical control [87].

Since 2020, seven RCTs have been published testing the analgesic properties of CBD: four testing the acute effects of CBD and three testing repeated dosing (2 and 12 weeks administration).

Looking first at the acute administration of CBD, no analgesic effects of a CBD solution (200, 400 or 800 mg) were observed in healthy volunteers (*n* = 17) who underwent a cold pressor test [88]. Another study in healthy volunteers (*n* = 20) also found no effect of CBD (800 mg in an oil based solution) on pain induced by intradermal electrical stimulation [89]. A single dose of CBD (150 mg in oil) also did not affect perceived soreness in untrained men (*n* = 13) after isokinetic exercises (exercise-induced muscle damage) [90]. Similarly, in patients with acute, non-traumatic low back pain (*n* = 100), CBD (400 mg in medium chain triglyceride oil) administration did not reduce pain scores or oxycodone use [91].

By contrast to the negative acute CBD pain studies, 4 weeks topical treatment with CBD in patients with symptomatic neuropathy (*n* = 29, mostly diabetic neuropathy) led to a significant reduction in intense pain, sharp pain, cold and itchy sensations in the CBD group when compared to the placebo group [33]. The product patients were using contained 250 mg of CBD per 3 fl. oz container and was advised to be applied up to 4 times per day to symptomatic areas. Another recent trial with a topical CBD product found 2 weeks treatment with topical CBD (6.2 mg/mL CBD with shea butter twice daily) resulted in improvements from baseline among patient-reported outcome measures compared to the control arm in patients (*n* = 18) with symptomatic thumb basal joint arthritis [32]. However, in patients with hand osteoarthritis or psoriatic arthritis (*n* = 129), oral treatment with CBD (20–30 mg/day) for 12 weeks did not affect pain intensity, sleep quality, depression or anxiety [92], perhaps reflecting too low a dose to be effective, or the difference between systemic and local administration of CBD.

In conclusion, the available clinical evidence does not support the acute analgesic effects of purified CBD. Two of three RCTs support the analgesic effects of topical CBD treatment to affected areas in neuropathy and arthritis. There is no evidence to date that oral CBD is an effective analgesic, although only one RCT has tested this, and used a low dose of CBD. However, this continues to be a strong area of research and there are more than 30 registered active trials investigating the use of CBD in chronic pain, endometriosis, musculoskeletal pain, back pain, dental pain, post-operative pain and opioid sparing. The data generated through these clinical trials should provide the necessary evidence as to whether CBD is an effective analgesic, and in which pain conditions.

## Purified CBD in movement disorders (Parkinson’s disease and Huntington’s disease)

The neuroprotective effects of CBD in multiple neurodegenerative conditions including the movement disorders Parkinson’s (PD) and Huntington’s (HD) diseases have been well studied preclinically, with CBD’s benefits mediated by its antioxidant and anti-inflammatory effects, and through direct or indirect activation of CB_1_, CB_2_, PPARγ and 5HT1A [93].

Small, uncontrolled pilot studies in patients with Parkinson’s have demonstrated improvements in dystonia over 6 weeks with oral CBD capsules (100–600 mg/day) [94] and in psychosis symptoms (Brief Psychiatric Rating Scale and the Parkinson Psychosis Questionnaire) over 4 weeks with oral CBD (*n* = 6, starting at 150 mg/day [95]). A small (*n* = 4), uncontrolled study by Chagas and colleagues also showed improvements in REM sleep behaviour disorder (RBD) in patients with Parkinson’s disease over 6 weeks (with individualised dosing in the 75–300 mg/day range) [82].

In contrast with the findings of the uncontrolled studies, two larger RCTs with 33 patients each examined the symptoms associated with Parkinsons, and did not report any differences in the frequency of nights with RBD symptoms [23] or severity of associated restless leg syndrome [96] with oral CBD doses of 75–300 mg over 14 weeks. In an RCT that included 24 patients, symptoms associated with anxiety, including anxiogenic tremor, were improved with a single 300 mg dose of CBD in a simulated public speaking test in patients with Parkinson’s disease (de Faria et al., 2020). Quality-of-life (PDQ-39) has also been shown to improve with the use of CBD (capsules, CBD 74 mg/day or 300 mg/day) daily over 6 weeks (*n* = 21) [24].

The effect of CBD on Huntington’s disease has only been examined in one RCT (*n* = 15), which did not show any difference in chorea severity between groups when CBD capsules were used at a dose of 10 mg/kg/day over 6 weeks [97].

In conclusion, although inconclusive, Parkinson’s patients may experience improvements in anxiety-related symptoms and overall quality-of-life. There is no evidence to support the use of CBD in Huntington’s disease. There are no registered trials investigating the potential of CBD in these indications, but the existing evidence would suggest this is worth pursuing.

## Purified CBD in the treatment of posttraumatic stress disorder (PTSD)

Preclinical evidence shows that pure CBD reduces symptoms in animal models of PTSD [67] and can confer additional benefits in combination with medications like sertraline [98], by similar mechanisms of action as have been identified for anxiety and psychosis,.

Three uncontrolled clinical studies have been published on the use of pure CBD in the treatment of PTSD. A case series in eleven patients found that oral CBD (mean total dose of 49 mg/day; 4 subjects received CBD via oral capsule only, 1 subject received CBD as an oral liquid spray and 6 patients received CBD either by capsules or spray) administered for 8 weeks decreased PTSD symptom severity as assessed by the PTSD Checklist for DSM-5 (PCL-5) [99]. A case report of a 10-year-old female survivor of acute sexual violence also showed that 5 months of CBD oil (25 mg orally at bedtime and 6–12 mg sublingual spray as required during the day) decreased anxiety and improved sleep quality and quantity [100]. Preclinical data suggests that CBD may prevent the development of PTSD [101]. This “prophylactic” use of CBD in PTSD has only been tested in one patient, a 15-year-old female survivor of acute sexual violence, but 7 days oral (capsule) dosing with 300 mg CBD did not prevent the onset of PTSD in this situation [102].

Several RCTs have examined the effects of a single dose of CBD in PTSD patients or healthy volunteers. Among 48 healthy volunteers, Das et al. (2013) examined the use of a single inhaled CBD dose (32 mg) and demonstrated that CBD attenuates fearful responses experienced during recall and reinstatement, suggesting the potentiation of consolidation of extinction memory, which is a key feature of PTSD. Bolsoni and colleagues reported that a single 300 mg CBD dose improved Visual and Analogical Mood Scale cognitive impairment factor scores for up to 1 week after administration in patients with PTSD (*n* = 33) [20]. In a separate report, the same investigators found that a single 300 mg CBD dose reduced subjective and physiological changes associated with traumatic recall in patients (*n* = 33) with a history of nonsexual trauma, but not in patients with sexual trauma [19].

Crippa and colleagues examined the effects of prolonged CBD treatment (150 mg twice daily for 28 days) in frontline health care professionals (*n* = 118) working with patients with COVID-19 (without pre-existing PTSD), on the basis that the risk of PTSD, emotional distress, depression and anxiety has been shown to be increased in that context. They found that, although PTSD symptoms did not differ between the treatment and controls arms, CBD did alleviate symptoms of anxiety, depression and emotional exhaustion [21]. A follow-up study found that the anxiolytic effects of CBD in frontline workers were maintained up to one month after discontinuation of treatment [103].

In conclusion, clinical evidence exists to support the use of single doses of CBD to reduce symptoms of PTSD. This is an area that continues to be researched in adults in PTSD (NCT05269459, NCT04197102), as an adjunctive to prolonged exposure therapy in PTSD (NCT03518801, NCT05132699), and in PTSD comorbid to traumatic brain injury (NCT04550377).

## Purified CBD for the management of substance abuse

There is preclinical evidence to show that CBD has anti-addictive properties and can reduce drug seeking behaviour for multiple substances including alcohol, opioids, cocaine and methamphetamine [104]. This has been investigated clinically against various substance abuse disorders, which are discussed below by drug.

In cannabis use disorder, one case report documents that oral CBD (300 mg/day, incrementing to 600 mg/day over 10 days) improved withdrawal, anxiety and dissociative symptoms seen with cannabis withdrawal syndrome [54]. A small uncontrolled study (*n* = 9) showed that inhaled CBD (mean 215.8 mg/day, with or without nicotine) administered via electronic cigarette led to a reduction in the overall consumption of cannabis by half over a period of 12 weeks in 30% of patients [105]. One RCT in cannabis use disorder has also found that CBD (400 or 800 mg per day over 4 weeks) can reduce urinary THC-COOH to creatinine concentrations, and increase cannabis abstinence more than placebo (*n* = 34) [36].

The evidence for the use of CBD in patients with cocaine use disorder is mixed. Although an RCT (*n* = 78) by Mongeau-Pérusse and colleagues did not show any difference in craving scores in response to drug cues with 800 mg/day oral CBD solution during 10-day detoxification and 12 weeks of subsequent follow-up [106], a nested substudy in 48 of the patients found that there was a reduction in inflammatory cytokines (IL-6, VEGF), monocytes and natural killer cells after CBD treatment, suggesting an anti-inflammatory response to CBD in these patients [35]. In patients with crack-cocaine dependence (*n* = 31), 10 days treatment with oral CBD (300 mg/day) did not affect measures of craving, anxiety, depression and sleep alterations [65].

One study has been carried out in patients with heroin use disorder (*n* = 42) which found that oral CBD (400 mg or 800 mg per day) over 3 days reduced craving and anxiety and improved heart rate and salivary cortisol responses in patients who were exposed to drug cues while abstinent [34].

In conclusion, evidence from a small number of trials shows that oral or inhaled CBD may help with abstinence in patients with cannabis or heroin abuse disorders and may reduce the inflammatory response seen when withdrawing from cocaine use. This indication continues to be well researched, with active trials with CBD in cannabis use disorder (NCT04105231), alcohol use disorder (NCT04873453, NCT05159830, NCT05387148), heroin use disorder (NCT04567784) and particularly in opioid addiction/sparing (NCT04308148, NCT03813095, NCT05299944, NCT03787628, NCT04982029, NCT04587791, NCT05076370).

## Purified CBD in gastrointestinal disorders

Preclinical evidence suggests that pure CBD could be useful in the treatment of a range of gastrointestinal disorders including the suppression of nausea and vomiting, intestinal hypermotility, intestinal inflammation, visceral pain, intestinal hyperpermeability and colon cancer [107, 108], with CB_1_, CB_2_, TRPV1, PPARα and 5HT1A implicated as the mechanisms of action.

The clinical evidence for CBD in gastrointestinal conditions comes from four RCTs, with mixed evidence of efficacy. Naftali and colleagues examined the effects of 8 weeks treatment with low-dose CBD (20 mg/day in an olive oil solution) in patients (*n* = 19) with Crohn’s disease. No difference was seen in the Crohn’s disease activity index between CBD and placebo groups at the end of treatment [109]. In healthy male volunteers (*n* = 30), a single dose of CBD (600 mg by capsule) significantly reduced the acute increase in intestinal permeability induced by aspirin [48]. Most recently, 4 weeks treatment with Epidiolex (20 mg/kg/day) did not improve any measured symptoms in patients (*n* = 48) with functional dyspepsia [110]. A chewing gum formulation of CBD (50 mg per gum for 3 weeks using ~ 6 gums per week) also had no impact on pain or quality of life scores in patients (*n* = 32) with irritable bowel syndrome (IBS) [111].

In conclusion, clinical data is currently lacking to support the use of purified CBD in gastrointestinal disorders. It is likely that the doses used in the IBD and IBS studies were too low, and therefore, further studies at clinically relevant doses are required to conclusively answer whether pure CBD could be beneficial in inflammatory bowel conditions, although there does not appear to be any active trials in this area.

## Purified CBD in autism

While there is limited preclinical research on the effects of CBD in autism spectrum disorder (ASD), there is growing real world evidence that CBD can be useful in the management of a number of symptoms of ASD, albeit usually using a CBD-dominant spectrum product containing THC and other phytochemicals [112, 113].

Focusing on studies where just purified CBD has been administered to autistic patients, there have been three RCTs. Two studies examined the effects of a single dose of CBD (600 mg in solution) on brain function in adult males with autism (*n* = 34) using functional magnetic resonance imaging and magnetic resonance spectroscopy. Both studies showed that 2 h after taking CBD, there are significant changes in neural connectivity and neurotransmitter (glutamate- gamma-aminobutyric acid (GABA)) signalling in areas of the brain implicated in ASD [28, 29]. While ASD symptoms were not measured in these studies, they demonstrate that pure CBD causes acute changes in brain activity in key areas of the brain controlling movement, language, social and visual processing in a relevant population.

A recent (*n* = 8) pilot study found that 8 weeks treatment with CBD (up-titrated to 20 mg/kg/day in two divided doses, with a maximum dose of 500 mg twice/day in an oil solution) improved the Aberrant Behaviour Checklist (ABC) score in children (age 8–16 years) with intellectual disability with severe behavioural problems [30]. Specific symptoms improved included irritability, social withdrawal, stereotypic behaviour and hyperactivity. While this was a feasibility study (well tolerated) and not powered to assess efficacy, it suggests that CBD may positively modify ASM symptoms as well as modifying brain function.

In conclusion, emerging evidence is promising that pure CBD can ameliorate symptoms associated with ASD. There are multiple active registered clinical trials (NCT03900923, NCT04520685, NCT04745026 and NCT04821856) that are investigating the effects of pure CBD in ASD (mostly in the form of Epidiolex) that will deliver data over the coming years in larger patient numbers.

## Purified CBD for the management of blood pressure

There is a strong preclinical evidence base that CBD has multiple effects on the cardiovascular system and has utility in the treatment of conditions including vascular dysfunction associated with diabetes, hypertension (particularly in stressful situations), myocardial infarction, cardiomyopathy, myocarditis and stroke [114–116].

There have been a limited number of studies that have attempted to translate this evidence to the clinic. In an RCT in healthy male volunteers, Jadoon and colleagues found that people (*n* = 9) who had taken pure CBD (600 mg by capsule) had lower blood pressure and a blunted blood pressure response to stress [47]. In a follow-up study, the ability of CBD to blunt the blood pressure response to stress was maintained in healthy volunteers (*n* = 26) after 7 days dosing with CBD (600 mg/day) [49]. This study also found that treatment with CBD increased internal carotid artery diameter, reduced arterial stiffness and improved endothelial function compared to day 1 in these healthy volunteers. A published clinical trial protocol suggests the concept that CBD has positive vascular effects is being tested in an RCT that is examining the influence of 5 weeks CBD administration on 24-h blood pressure, arterial stiffness, CBD and vascular health biomarkers, inflammation, heart rate variability in individuals with mild or moderate hypertension [117].

In conclusion, preclinical and healthy volunteer data suggest there is a positive impact of pure CBD on the blood pressure response to stress and on vascular health. Ongoing research will validate whether this effect is observed in a clinically relevant population, although this is an area with limited active research.

## Purified CBD in the treatment of cancer

The anti-tumoural effects of CBD have been extensively studied. A systematic review of preclinical literature identified 83 studies in human cancer cell lines showing that pure CBD inhibits cell viability and proliferation, inhibits cancer cell migration, is anti-inflammatory and reduces angiogenesis and metastasis in multiple cancer types in vitro and in vivo [118].

No RCTs with purified CBD in cancer progression in patients have been conducted. However, there are three peer-reviewed publications documenting the cases of cancer patients who have observed positive effects of CBD on cancer progression. Retrospective case reports of patients (*n* = 17 across two publications) with brain tumours (glioblastoma multiforme and high-grade glioma) found that daily treatment with CBD (between 100 and 600 mg/day by capsule) was associated with longer survival rates than was expected in this cohort [119, 120]. In 2018, an analysis of 119 cancer patients with solid tumours from a private clinic claimed that 92% of cases showed some kind of clinical response (reduced circulating tumour cells or a reduction in tumour size) to low-dose purified CBD (average 10 mg twice daily) [121].

In conclusion, preclinical and published case reports suggest an anti-tumoural effect of pure CBD, but this is yet to be tested through controlled clinical trials. Several registered trials are examining the utility of CBD in ameliorating symptoms of cancer including anxiety (NCT04482244) and pain (NCT04754399 and NCT05388058), but not on tumour progression.

## Purified CBD in COVID

With the outbreak of COVID-19, many postulated that CBD could potentially be a therapy to reduce the acute or chronic symptoms of the virus. One RCT has since been published in this area (the CANDIDATE Study). This randomised, double-blind, placebo-controlled trial examined the use of oral CBD (300 mg/day) over 2 weeks in 91 patients with mild-to-moderate COVID-19 symptoms. There was no between-group difference in the primary outcome of time to symptom resolution, or secondary outcomes, including inflammatory markers, emotional symptom scales, viral load, smell test or side effects. Thus, current evidence does not support the use of CBD to treat non-severe COVID-19 [21].

## Purified CBD for the management of smoking

The potential use of CBD for managing substance abuse has already been discussed, and some studies have also examined whether CBD could be used to reduce nicotine intake. A small (*n* = 24) randomised pilot study in 2013 demonstrated that inhaled CBD (400 µg doses over 1 week) reduced the consumption of cigarettes by 40% in the treatment group compared with placebo [38]. Hindocha and colleagues also showed that a single 800 mg oral dose of CBD reduced the pleasantness and salience of cigarette smoking cues compared with placebo in an RCT of 30 patients [37]. These studies support the use of, and further research into, CBD as a component of smoking cessation programs. Only one registered trial appeared to be continuing this research (NCT05445804).

## Purified CBD in the treatment of type 2 diabetes

Preclinical studies have shown that pure CBD treatment can reduce pancreatic inflammation and the incidence of type 1 diabetes [122, 123], improve metabolic dysfunction (increased insulin, reduced blood glucose, lower fructosamine, lower lipid levels and total cholesterol levels ) in type 2 diabetes [124], reduce diabetic neuropathy [125] and is neuroprotective in middle aged diabetic rats [126]. However, the only RCT has examined the effects of pure CBD (100 mg twice daily for 13 weeks) in patients with type 2 diabetes did not see any positive metabolic effects of CBD [127]. At this dose, CBD failed to affect the primary endpoint (HDL cholesterol) or other markers of metabolic function (glycaemic control, lipid profile, insulin sensitivity, body weight, liver triglyceride content, adipose tissue distribution, appetite, markers of inflammation, blood pressure, markers of vascular function, and circulating endocannabinoids). Within the CBD group, there was a significant decrease in the adipokine resistin, and an increase in the gut hormone glucose-dependent insulinotropic peptide (GIP), although the significance of these changes is unknown given the lack of response against other metabolic parameters. A registered trial continues to investigate the impact of CBD on glucose tolerance in volunteers with a BMI greater than 25 m^2^ (NCT05285449), although using a low dose that is unlikely to show efficacy (30 mg twice per day). Given the preclinical evidence, it is likely that the dose of CBD used in the RCT was not sufficient to see beneficial effects, and further research is required to test the use of CBD in diabetes, either for direct effects on metabolic function, or for associated problems such as vascular dysfunction, cardiopathy, neuropathy or neuroprotection.

## Purified CBD in graft versus host disease

Because of the anti-inflammatory and immunosuppressive effects of CBD, a single prospective trial (*n* = 48) examined the effect of CBD on the incidence and severity of graft-versus-host disease (GVHD) after allogeneic haematopoietic cell transplantation [128]. In this study, the majority (79%) of patients had acute leukaemia or myelodysplastic syndrome. Among patients receiving 150 mg CBD twice daily from 7 days pre-transplantation to 30 days post-transplantation, in addition to standard GVHD prophylaxis, the authors observed a reduction in grades II to IV acute GVHD compared with controls. The authors also noted that no patient developed GVHD while taking CBD. Although data are limited to a single study, CBD may have some benefits in terms of reducing GVHD for patients undergoing allogeneic hematopoietic cell transplantation. The status of several registered trials in this area is unknown (NCT01596075, NCT01385124, NCT02478424), although there is still one active trial (NCT03840512).

## Purified CBD in the treatment of intraocular pressure

There is a small body of literature that has assessed the effects of cannabinoids (mainly THC) in the eye, with a limited number of studies showing benefit of CBD in models of diabetic retinopathy and corneal hyperalgesia [129]. There is only one clinical trial in this field that examined the effects of THC (5 mg) or CBD (20 and 40 mg, acute dose, sublingual) in patients with ocular hypertension or early primary open angle glaucoma [130]. Although THC reduced intraocular pressure 2 h after administration, CBD did not reduce intraocular pressure at any time, and in fact the higher dose produced a transient increase in intraocular pressure at 4 h.

## Effective doses and the therapeutic ranges

**Fig. 2:**
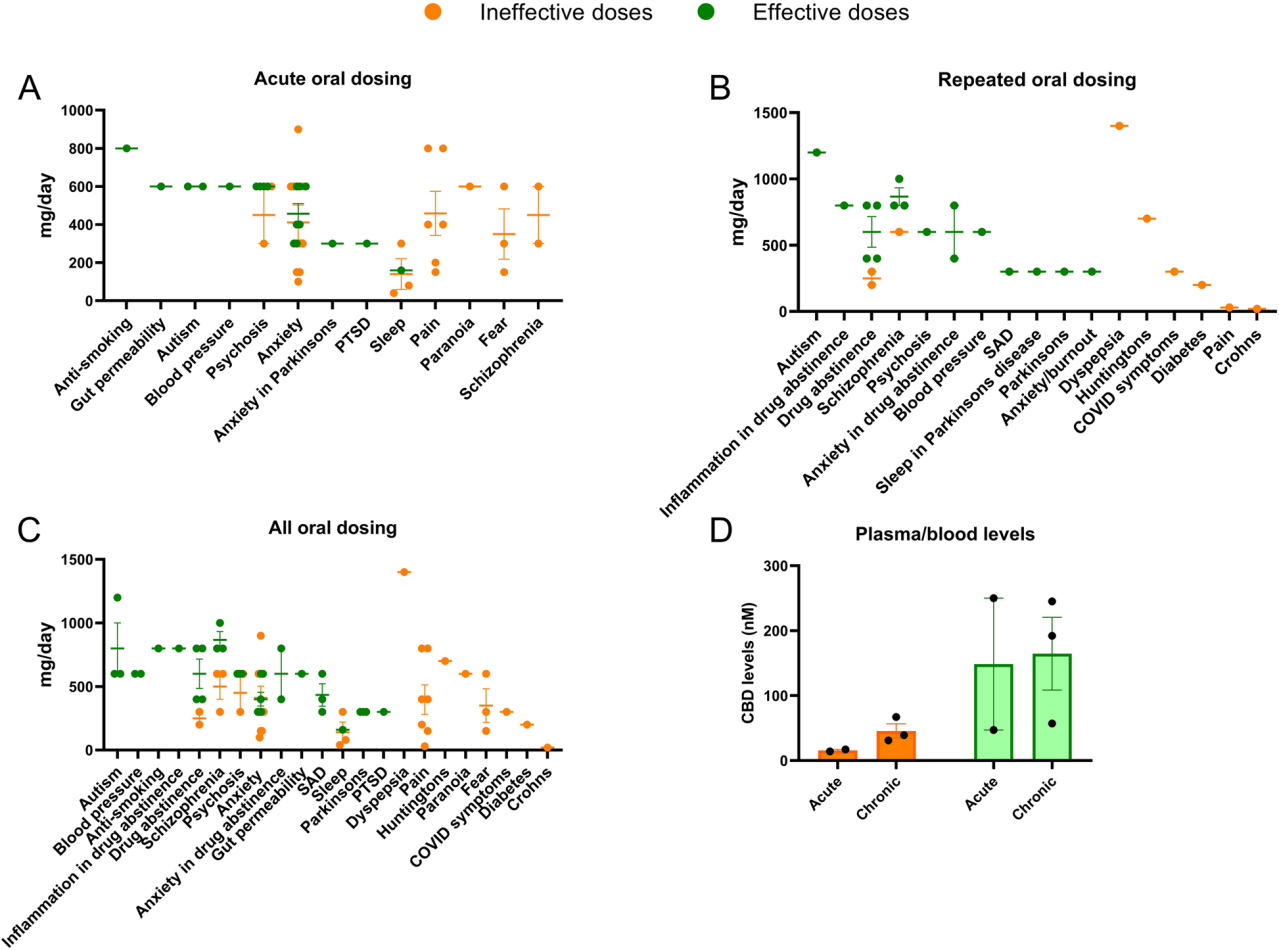
The effective (green) and ineffective (orange) doses of purified CBD (mg/day) tested through randomised controlled trials (RCTs) in the acute setting **A** and with repeated dosing **B** across different indications (including healthy volunteer studies). **C** A summary of all doses in all contexts tested though RCTs. **D** Plasma or blood levels of CBD (where tested) in acute dosing or repeated doing clinical studies with purified CBD that reported positive (green) or negative (orange) outcomes. Each dot represents an RCT

A big question in the clinical use of CBD is how much is enough? To answer this, we have looked at the randomised controlled studies with CBD, as uncontrolled studies tend to underestimate effective doses. Single doses of CBD that have a significantly different biological impact compared to placebo are in the range 160 mg to 800 mg (see Fig. 2A). In RCTs where a positive effect of repeated CBD treatment (on any endpoint) in patients has been recorded compared to placebo, the daily dosing was in the range of 300 to 1200 mg/day (see Fig. 2B). In RCTs where no impact of CBD (on any endpoint) was observed, dosing was in the range of 20 to 800 mg. While there is considerable overlap here, studies that were positive did tend to use a higher mean dose of CBD. Negative trials with CBD could be the result of insufficient dosing or could also be due to poor clinical trial design or the wrong patient populations. Thus, it is sometimes difficult to tell from negative studies whether CBD is ineffective in that particular condition or whether the doses tested were too low. This is confounded by the fact that the oral bioavailability of CBD is low, and only about ~ 10% of the drug will enter circulation [131], and by the fact that many published studies have only looked at a single dose of CBD.

Unfortunately, very few studies have measured the plasma or tissue levels of CBD as a guide to the therapeutic range of CBD, but where reported, the mean plasma levels of CBD in studies that report a significant effect of CBD on endpoints measured (in either acute or repeated dosing studies) are higher than those that do not (~ 150 nM versus 30 nM respectively, see Fig. 2D). For reference, the mean serum concentration of CBD is ~ 125 ng/ml (~ 350 nM) in patients with epilepsy [8]. CBD has dozens of molecular targets with various potency [50], mostly low, and the therapeutic target levels for CBD probably vary between conditions depending on the most important molecular targets in that particular condition.

## Conclusions

**Fig. 3:**
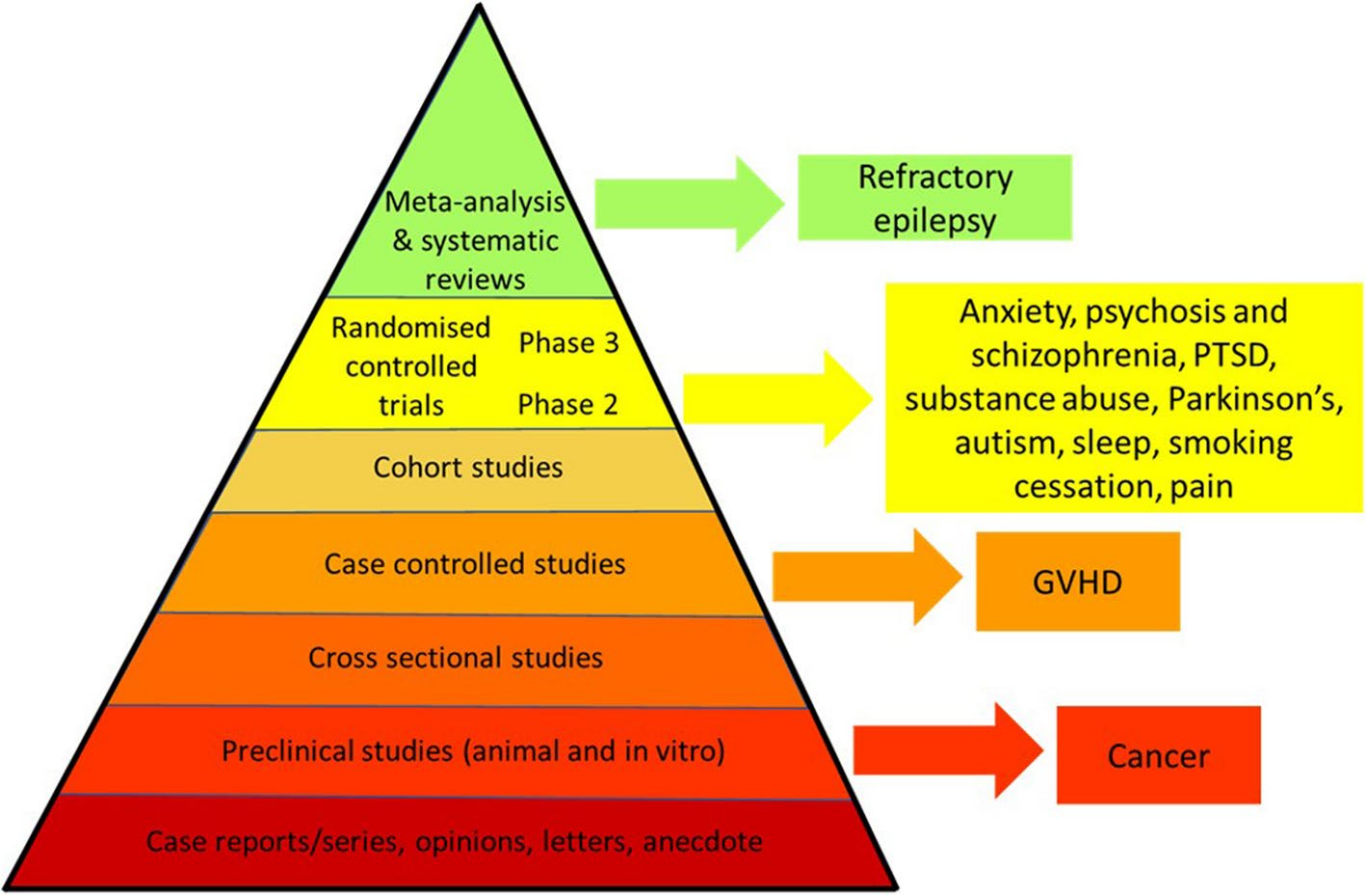
The current state of affairs with regards to the therapeutic utility of purified CBD according to the hierarchy of evidence. GVHD, graft-versus-host disease; PTSD, post-traumatic stress disorder

Purified CBD is licensed for the treatment of some epilepsy conditions, but mounting evidence suggests that this drug could be used for the treatment of other conditions. This evidence is strongest for psychiatric conditions including anxiety, psychosis and schizophrenia, PTSD and substance abuse, which is underpinned by preclinical data implicating many of the same mechanisms of actions (for example, cannabinoid, serotonin, glycine and GABA receptors). However, this evidence is based on small, proof of concept clinical studies with low patient numbers and so considered weak in the hierarchy of evidence. The strength of evidence for CBD across multiple conditions is presented in Fig. 3. Many of these indications continue to be investigated in registered clinical studies, so the evidence base may be strengthened in the short to medium term. By contrast, there are far fewer studies that have investigated CBD in non-neurological conditions despite a wealth of preclinical evidence of CBDs benefits in conditions such as cardiometabolic disorders and cancer that warrant testing clinically. There is also little active research in this area, so limited data will be emerging in these indications. Future clinical studies should look at the efficacy of ranges of doses of CBD in larger patient numbers and in both genders, establishing target plasma levels for CBD across various indications. Future studies could then establish whether the efficacy of purified CBD can be enhanced with the addition of other phytochemicals, and how CBD affects the efficacy of concomitant treatments.

## Data Availability

Not applicable.
